# On the Analysis of a Repeated Measure Design in Genome-Wide Association Analysis

**DOI:** 10.3390/ijerph111212283

**Published:** 2014-11-28

**Authors:** Young Lee, Suyeon Park, Sanghoon Moon, Juyoung Lee, Robert C. Elston, Woojoo Lee, Sungho Won

**Affiliations:** 1 The Center for Genome Science, Korea National Institute of Health, KCDC, Osong 361-951, Korea; E-Mails: lyou7688@gmail.com (Y.L.); sooyeon1002@gmail.com (S.P.); jiap72@hanmail.net (S.M.); jylee_monte@naver.com (J.L.); 2 Department of Applied Statistics, Chung-Ang University, Seoul 156-756, Korea; 3 Department of Epidemiology and Biostatistics, Case Western Reserve University, Cleveland, OH 44106, USA; E-Mail: robert.elston@cwru.edu; 4 Department of Statistics, Inha University, Incheon 402-751, Korea; 5 Department of Public Health Science, Seoul National University, Seoul 151-742, Korea

**Keywords:** longitudinal data, cross-sectional data, Korean Association Resource (KARE) cohort, Health Examinee (HEXA) cohort

## Abstract

Longitudinal data enables detecting the effect of aging/time, and as a repeated measures design is statistically more efficient compared to cross-sectional data if the correlations between repeated measurements are not large. In particular, when genotyping cost is more expensive than phenotyping cost, the collection of longitudinal data can be an efficient strategy for genetic association analysis. However, in spite of these advantages, genome-wide association studies (GWAS) with longitudinal data have rarely been analyzed taking this into account. In this report, we calculate the required sample size to achieve 80% power at the genome-wide significance level for both longitudinal and cross-sectional data, and compare their statistical efficiency. Furthermore, we analyzed the GWAS of eight phenotypes with three observations on each individual in the Korean Association Resource (KARE). A linear mixed model allowing for the correlations between observations for each individual was applied to analyze the longitudinal data, and linear regression was used to analyze the first observation on each individual as cross-sectional data. We found 12 novel genome-wide significant disease susceptibility loci that were then confirmed in the Health Examination cohort, as well as some significant interactions between age/sex and SNPs.

## 1. Introduction

Disease prognosis and personalized medicine require the identification of genetic and non-genetic risk factors and, with the rapid improvement of genotyping technology, more than ten thousand genome-wide association studies (GWAS) have been conducted to discover disease susceptibility loci. Since the first such successful study in 2005 [1], more than ten thousand disease susceptibility loci have been successfully identified and these findings have improved our understanding of the genetic background of human diseases. However, in spite of these successes in GWAS, causal genetic variants identified by GWAS explain only a small proportion of the heritability [2,3]. Various reasons, including the common disease/rare variant hypothesis, have been put forward to explain this so-called missing heritability [4]. However, the missing heritability is partially attributable to a large number of false negative findings induced by insufficient sample sizes when controlling for multiple testing [5], and various strategies, such as GWAS using multiple phenotypes or longitudinal data [6,7], have been considered to overcome these problems. The analysis of multiple phenotypes can suffer from their inherent heterogeneity, but the analysis of the multiple measures of the same phenotype provided by longitudinal data may avoid this issue and, if measurement errors are substantial, GWAS with longitudinal data can be expected to mitigate the sample size problem.

Even though there are few GWAS using longitudinal data [8,9,10], compared to cross-sectional data longitudinal data have various useful features. First, although phenotyping is sometimes more expensive than the cost of genotyping, in those situations where the cost of genotyping is more expensive than that of phenotyping, repeated measurements at different time points have the virtual effect of enlarging the sample size. Second, with longitudinal data, the total phenotypic variance can be decomposed into among-subject and within-subject components. Third, phenotypes at different time points can be compared with baseline phenotypes and any confounding effect due to age can be prevented. Fourth, the onset of some diseases is sometimes affected by genetic variants, and gene × age interaction can be estimated with better accuracy. In this report, we conducted GWAS with longitudinal data in the Korean Association Resource (KARE) cohort. Phenotypes in the KARE cohort were measured every two years from 2001 to 2005, and we performed GWAS for eight phenotypes with three repeated measurements: systolic blood pressure (SBP), diastolic blood pressure (DBP), fasting plasma glucose (GLU0), 2-h OGTT glucose (GLU120), height, body mass index (BMI), high-density lipoprotein (HDL) and aspartate aminotransferase (AST). Results from the longitudinal GWAS were compared with those from GWAS using cross-sectional data, and our results showed that GWAS using longitudinal data provided more significant results. We identified 12 novel variants associated with phenotypes: rs11067763 (near MED13L) for DBP; rs12991703 (near MARCO) and rs7197218 (in XYLT1) for GLU0; rs17178527 (in AK097143) for BMI; rs12292858 (in SIK3), rs11066280 (in HECTD4) and rs183786 (near ALDH1A2) for HDL; and rs10849915 (in CCDC63), rs3782889 (in MYL2), rs12229654 (near MYL2-CUX2), rs11066280 (in HECTD4) and rs2072134 (in OAS3) for log-transformed AST. These variant associations were found to replicate in the Health Examinee (HEXA) cohort and thus illustrate the practical value of a longitudinal data analysis.

## 2. Materials and Methods

### 2.1. The Korean Association Resource (KARE) Cohort

The KARE cohort consists of a total of 10,038 individuals (5018 and 5020 individuals from Ansung and Ansan, respectively). Participants ranged from 40 to 69 years old, and their phenotypes were consecutively measured with two-year intervals from 2001 to 2005. Among the 10,038 participants, 10,004 individuals were genotyped for 500,568 SNPs with the Affymetrix Genome-Wide Human SNP array 5.0. Individuals and SNPs with call rates less than 95% were excluded from the analysis. SNPs with *p*-values for Hardy-Weinberg equilibrium (HWE) less than 10^−6^, or with minor allele frequencies (MAF) less than 0.01, were eliminated. Furthermore, individuals with tumors, gender inconsistencies, or whose heterozygosity rates were more than 30%, or identity in state (IBS) more than 0.8, were excluded from the analysis [11]. In total, 8842 individuals with 352,228 SNPs were available at the baseline time-point. At the second and third time-points, there were some missing phenotypes, and phenotypes for 7568 and 6675 individuals, respectively, were available.

### 2.2. The Health Examinee (HEXA) Cohort

Independent individuals in the HEXA cohort were from a second population based cohort sample provided by the Health study. This study combines subjects from the Wonju, Pyeong Chang, Gangneung, Geumsan, and Naju regional cohorts in Korea. There are 120,000 participants in the HEXA cohort, 4302 of whom, between 40 and 69 years old, were randomly selected for genotyping with the Affymetrix Genome-Wide Human SNP array 6.0. Individuals in the HEXA cohort were used to replicate the significant findings found in the KARE cohort, and individuals with tumors, large heterozygosity rates, gender inconsistencies, evidence of non-Asian ancestry, or whose IBS was more than 0.8 or call rates were less than 95%, were excluded from analysis [12]. SNPs with *p*-values for HWE less than 10^−6^, genotype call rates less than 95%, or MAF less than 0.01, were excluded, and the remaining SNPs for 3703 individuals were used for the analysis. In particular, the HEXA cohort is a cross-sectional study, and if there are some SNPs which have a progressing effect on phenotypes, results from HEXA and KARE cohort could be heterogeneous.

### 2.3. Sample Size Calculation for a Longitudinal Study

Sample sizes required to achieve 0.8 power at the 10^−8^ significance level were calculated in both the presence and absence of population substructure. We denote the number of individuals and measurements for each individual by *n* and *t*, respectively. We assume that *t* is same for all individuals. The additively coded value of the genotype for individual *i* is denoted by *X_i_*. We assume Hardy-Weinberg equilibrium and each individual’s genotype is assumed to follow a trinomial distribution. The effect of the disease allele is assumed to be β. We assume a matrix of environmental effects that does not contain time-varying covariates for individual *i*, denoted by **Z*_i_***, and its coefficient is assumed to be the column vector **α**. The columns of **Z*_i_*** denote each covariate. The phenotype for individual *i* at time-point *j* is denoted by *Y_ij_*, and the corresponding *t*-dimensional vector by **Y***_i_*. Letting **1***_t_* be the *t*-dimensional column vector with elements 1, **Y***_i_* was assumed to be:

(1) $${\text{Y}}_{i}={\text{Z}}_{i}\text{α}+(X_{i}\beta )1_{t}+g_{i}1_{t}+c_{i}1_{t}+{\text{ε}}_{i},\;c_{i}\sim N(0,\sigma_{c}^{2}),{\text{ε}}_{i}\sim MVN(0,\sigma_{\varepsilon }^{2}\text{I})$$

where ***c**_i_* indicates the random effect which explains the similarity of repeated measurements for each individual attributable to a non-polygenic effect. We denote *σ_p_*^2^ = *σ_g_*^2^ + *σ_c_*^2^ + *σ_ε_*^2^ and *ρ* = *σ_c_*^2^/*σ_p_*^2^. Thus ρ measures the proportion of the variance of *c_i_* relative to the phenotypic variance. In particular, we included in the model to provide a polygenic effect for individual *i*, and assumed as an approximation $\text{g}=(g_{1},g_{2},\ldots ,g_{n})\prime$ follows the multivariate normal distribution with mean vector **0** and variance-covariance matrix ${\text{σ}}_{g}^{2}\text{Φ}$ where **Φ** corresponds to the genetic relationship matrix. If ${\text{σ}}_{g}^{2}$ $\sigma_{g}^{2}$ is 0, the correlation matrix **R** of **Y***_i_* becomes compound symmetric. The null hypothesis *H*_0_ is β = 0, and β is assumed to be β*_a_* under the alternative hypothesis.

For sample size calculation, we assumed that there is no covariate effect other than the genotypes, and then we could assume that the environmental variable ***Z**_i_* is **1***_t_*. Then if we let *π_l_* = *P*(*X_i_* = δ*_l_*, *Z_i_* = 1) = *P*(*X_i_* = δ*_l_*) for the coded genotype δ*_l_* where δ_1_ = 0, δ_2_ = 1 and δ_3_ = 2, and *K* = *π*_1_(*π*_2_ + 2*π*_3_)^2^ + *π*_2_(*π*_3_ − *π*_1_)^2^ + *π*_3_(1 + *π*_1_ − *π*_3_)^2^, the required sample size for 1 − ϕ power at the significance level *α* can be derived to be:

(2) $$n_{\text{α}}=\frac{\left(z_{1-\frac{\text{α}}{2}}+z_{1-\phi }\right)\sigma_{p}^{2}}{K{\text{β}}_{\alpha }^{2}}\left(\frac{1+(t-1)\text{ρ}}{t}\right)$$

(see the Appendix for the detailed derivation). Liu and Liang [13] derived the required sample sizes when *X_i_* is binary and our results are based on their derivations. We extend their result to *X_i_* with arbitrary many levels. For *n_α_*, we assume that σ*_c_*^2^ + σ *_ε_*^2^ = 1 and:

(3) $$\frac{2{\text{β}}^{2}p\left(1-p\right)}{2{\text{β}}^{2}p\left(1-p\right)+{\text{σ}}_{c}^{2}+{\text{σ}}_{\varepsilon }^{2}}=0.005$$

In the presence of population substructure, σ*_g_*^2^ is assumed to be larger than 0. The required sample size cannot be directly calculated, and we calculated *n_a_* by simulation studies based on a Monte Carlo method. In our simulations, we assumed that σ*_g_*^2^ + σ*_c_*^2^ + σ *_ε_*^2^ = 1 and the effect of disease the allele, β, was calculated with the following assumptions:

(4) $$\frac{2{\text{β}}^{2}p\left(1-p\right)}{2{\text{β}}^{2}p\left(1-p\right)+{\text{σ}}_{g}^{2}+{\text{σ}}_{c}^{2}+{\text{σ}}_{\varepsilon }^{2}}=0.005\;\text{and}\;h^{2}=\frac{2{\text{β}}^{2}p\left(1-p\right)+{\text{σ}}_{g}^{2}}{2{\text{β}}^{2}p\left(1-p\right)+{\text{σ}}_{g}^{2}+{\text{σ}}_{c}^{2}+{\text{σ}}_{\varepsilon }^{2}}=0.3$$

where these equations indicate that the proportion of genetic variance explained by the causal genotype is 0.005 and heritability is 0.3. For convenience, **Φ** was assumed to be a compound symmetric matrix with off-diagonal elements 0.1. The off-diagonal elements are asymptotically equivalent to twice the kinship coefficient between two individuals, and 0.1 means that the individuals are genetically remote relatives.

### 2.4. Genome-Wide Association Studies (GWAS) Using Longitudinal and Cross-Sectional Data

In the Korean Association Resource (KARE) data, eight phenotypes (SBP, DBP, GLU0, GLU120, height, BMI, HDL, and AST) were observed every two years from 2001 to 2005, so that three observations were available for each phenotype. Genotypes and phenotypes for 8842 individuals were initially available. However, there were some missing phenotypes for follow-up observations, and only 7568 and 6675 individuals were observed for the second and third time-points, respectively. Reasons for dropout were not known, but may include death, immigration, and non-response.

GWAS using longitudinal data were performed by generalized least squares using the nlme package in the R software. The phenotype for individual *i* is denoted by **Y***_i_* which is a three dimensional vector. The matrix **Z***_i_* indicates a covariate vector for environmental effects, including the intercept as the first column, and sex, age and age^2^ at the first time-point were included as covariates for all eight phenotypes. In particular, weight is known to be related to glucose levels, and thus it was included as an additional covariate for the GWAS of GLU0 and GLU120 [14,15]. The coefficient vector of **Z***_i_* is denoted by a vector **α**. The effect of the time interval can be understood as the effect of aging, and it was denoted by the vector **w**. Here, **w** is a three-dimensional row vector and its coefficient is η. The population substructure between individuals was adjusted for with the EIGENSTRAT approach [16] and the remaining potential bias unadjusted by EIGENSTRAT was further adjusted for by the genomic control method [17]. In particular, the IBS matrix is often better than the identity-by-descent matrix for capturing the long-distance relationships that result from variations at the population level [18] and we used the IBS matrix for EIGENSTRAT. The first five principal component (PC) scores accounted for 75% of the variation in the IBS matrix, and they were used as covariates to adjust for any population substructure. The PC score vectors for individual *i* and its coefficient vector are **PC***_i_* and **γ**, respectively. The additively coded value of the genotype for individual *i* is denoted by *X_i_*. The effect of the disease allele is assumed to be β. The variance-covariance matrix for **ε***_i_* is denoted **Σ** and assumed to be an unstructured symmetric matrix. For longitudinal analysis, our final model is:

(5) $${\text{Y}}_{i}={\text{Z}}_{i}\text{α}+(X_{i}\beta )1_{3}+PC_{i}\gamma +\text{w}\eta +{\text{ε}}_{i}$$

where w=(0,2,4)′, ${\text{ε}}_{i}=({\text{ε}}_{i1},{\text{ε}}_{i2},{\text{ε}}_{i3})\prime$ is distributed as MVN(0,Σ) for *i* = 1,2, …, 8842.

Furthermore, we conducted GWAS using cross-sectional data for comparison with the GWAS using longitudinal data. For this we took the phenotypes at the first-time point in the KARE cohort, and the GWAS were conducted with linear regression. For the cross-sectional analysis, we used the same covariates except for time interval, with the linear model:

(6) $$Y_{i}={\text{Z}}_{i}{\text{α}}_{i}+X_{i}\beta \sum_{k=1}^{5}PC_{ik}\gamma_{k}+\varepsilon_{i}$$

where ${\text{ε}}_{i}$ is distributed as $N(0,{\text{σ}}^{2})$ for *i* = 1,2, …, 8842. The results from longitudinal and cross-sectional data were compared.

We tested whether there exist any interaction effects between our significant findings and environmental variables by adding interaction terms as covariates. We considered age, sex and time interval as environmental variables, and their statistical interaction with the SNPs were tested. Significant results were further tested for replication in the HEXA cohort (other than time interval, because the HEXA cohort only has cross-sectional data). Finally, we tested all the significant findings from the KARE cohort, as a discovery dataset, in the HEXA cohort, as a replication dataset.

## 3. Results

### 3.1. Sample Size for a Longitudinal Cohort Design

**Figure 1:**
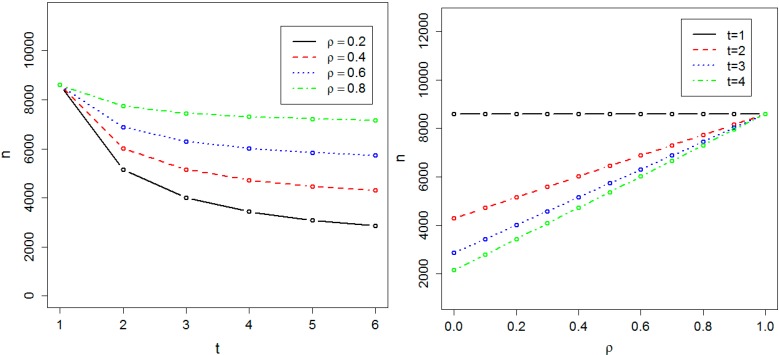
Required sample size in the absence of population substructure. The sample size is indicated by *n*. The required sample size to achieve 0.8 power at the significance level α = 10^−8^ has been calculated as a function of *t*, the number of time points, and ρ, the correlation between measurements at two different time-points.

**Figure 2:**
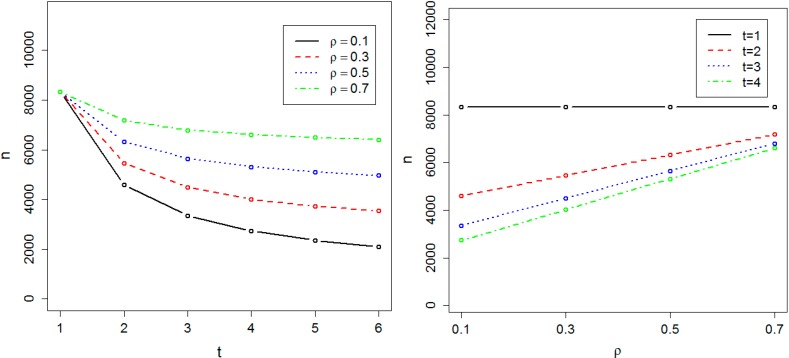
Required sample size in the presence of population substructure. The sample size is indicated by n. The required sample size to achieve 0.8 power at the significance level α = 10^−8^ has been calculated as a function of t, the number of time points, and ρ, the correlation between measurements at two different time-points.

We calculated the sample size required to achieve 0.8 power at the genome-wide significance level α = 10^−8^ in both the absence and presence of population substructure. Figure 1 and Figure 2 respectively show the required sample size *n_α_*, in the absence and presence of population substructure, as a function of the number of time points *t* and the common correlation *ρ* between phenotype measures on the same person. We found the required sample size *n_α_* is proportionally related to *t* and inversely related to ρ. Sample size is minimized for small *ρ* and large *t*, and the effect of *t* on sample size is maximal when ρ = 0. These results illustrate the practical efficiency of GWAS with longitudinal data. For instance, if ρ = 0.4 and *t* = 3, then 5158 individuals are sufficient to achieve 0.8 power at the genome-wide significance level and, compared to cross-sectional data, genotyping costs for 3438 individuals can be saved *vs.* the cost of obtaining 6878 phenotypes.

### 3.2. Genome-Wide Association Studies (GWAS) with Longitudinal Data for Eight Phenotypes

**Figure 3:**
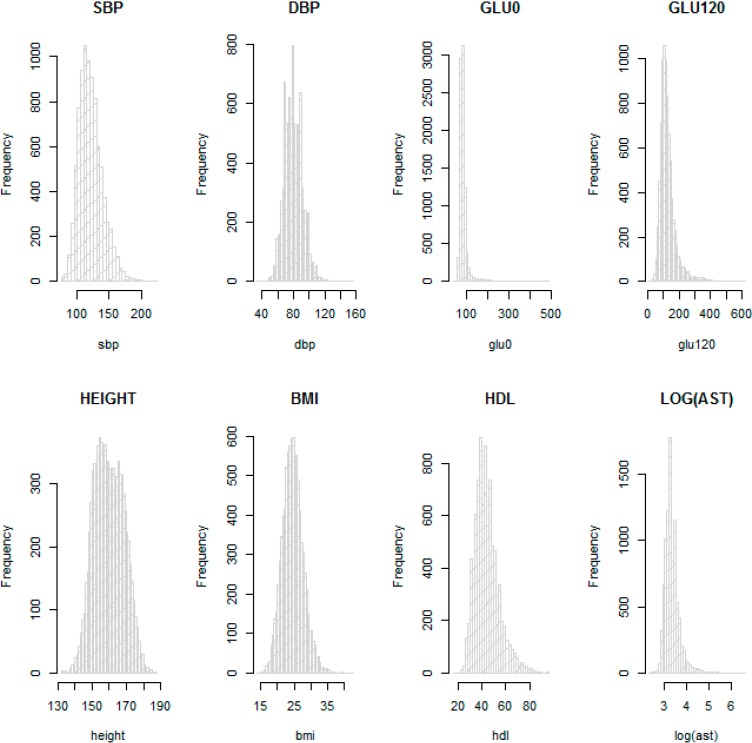
Histograms for SBP, DBP, GLU0, GLU120, HEIGHT, BMI, HDL and log AST in the KARE cohort.

Table 1 and Table 2 provide descriptive statistics for sex, age and other available phenotypes from the KARE and HEXA cohorts. These show that the distributions of phenotypes are similar in the HEXA cohort the KARE cohort at each time point. We checked the normality of the eight phenotypes with histograms. In particular, AST was not normally distributed and so was log-transformed. Figure 3 shows that log-transformed AST and the other seven phenotypes on the original scale are about normally distributed, so these were used for the GWAS.

**Table 1:** Sample sizes for Korean Association Resource (KARE) cohort.

Time Point	KARE					
	1		2		3	
	N(Ansan/Ansung)	Age(s.d)	N(Ansan/Ansung)	Age(s.d)	N(Ansan/Ansung)	Age(s.d)
Male	2374/1809	51.78(8.79)	1967/1642	53.71(8.82)	1758/1424	55.58(8.71)
Female	2263/2396	52.61(9.02)	1764/2213	54.60(8.99)	1543/1950	56.48(8.90)
Total	4637/4205	52.22(8.92)	3731/3855	54.18(8.92)	3301/3374	56.05(8.82)

**Table 2:** Descriptive statistics for eight quantitative phenotypes examined in the Korean Association Resource (KARE) and Health Examinee (HEXA) cohorts.

Time Point	KARE Cohort						HEXA Cohort	
	1		2		3			
Phenotype	Mean(s.d)	*N*	Mean(s.d)	*N*	Mean(s.d)	*N*	Mean(s.d)	*N*
SBP	121.65(18.61)	8842	118.6(17.3)	7504	116.6(16.62)	6646	121.69(14.36)	3703
DBP	80.26(11.46)	8842	78.49(10.96)	7504	77.69(10.25)	6646	77.05(9.84)	3703
GLU0	87.66(21.88)	8581	92.74(15.14)	6688	92.31(15.15)	5985	94.10(24.56)	3703
GLU120	126.76(51.03)	8387	125.77(41.59)	4865	134.07(50.56)	5985	Not available	
height	160(8.67)	8842	159.93(8.74)	7461	159.95(8.76)	6596	161.49(8.10)	3703
BMI	24.6(3.12)	8838	24.59(3.09)	7456	24.52(3.05)	6596	23.96(2.88)	3703
HDL	44.65(10.09)	8841	46.27(9.90)	7495	44.04(10.25)	6640	54.60(13.27)	3703
AST	29.81(18.41)	8841	24.67(14.95)	7495	25.87(19.02)	6640	24.51(12.94)	3703

The results of the GWAS with longitudinal data in the KARE cohort were compared with the results from GWAS using cross-sectional data. For the cross-sectional data we used the phenotypes at the first time-point, applying linear regression. Population substructure was adjusted for with the EIGENSTRAT method, and five principal component (PC) scores were included as covariates in both the longitudinal and cross-sectional data analyses. We found that five PC scores explain roughly 75% of the kinship matrix, and Table 3 shows the estimated variance inflation factors, λ, obtained by genomic control [17]. The estimated variance inflation factors from the longitudinal data analyses were always slightly larger than those from the cross-sectional data analyses, which suggests that longitudinal data analysis tends to be more sensitive to population substructure. Even though more detailed sensitivity analyses are necessary to confirm whether the model assumption for longitudinal data analyses are satisfied, our findings are probably not affected by population substructure because the quantile-quantile (QQ) and Manhattan plots for the eight phenotypes in Supplementary Figures 1–4 consistently show the validity of our analysis.

**Table 3:** Inflation factors by genomic control.

Phenotype	Cross-Sectional Data	Longitudinal Data
SBP	1.040	1.051
DBP	1.028	1.053
GLU0	1.022	1.026
GLU120	1.026	1.037
height	1.069	1.071
BMI	1.046	1.052
HDL	1.034	1.039
log AST	1.023	1.030

We calculated the correlations between the different time-points for each phenotype and they are presented in Table 4. The correlations for height and BMI are usually very large and those for log AST are the smallest. Therefore, the improvement in power on using longitudinal data is expected to be the most substantial for log AST, and it seems to be almost negligible for height and BMI. Table 5 and Table 6 show the results from GWAS using longitudinal data and cross-sectional data in the KARE cohort, and the significant results were further tested in the HEXA cohort. The cross-sectional data for the KARE cohort are the first measurements for each individual in the longitudinal data. Cross-sectional and longitudinal data in the KARE cohort were analyzed with linear regression and a linear mixed model, respectively, and SNPs with *p*-values from either the cross-sectional or longitudinal data analysis less than 10^−6^ were selected for the replication studies. For the discovery analyses, the genome-wide significance level by Bonferroni correction is 1.4E − 07. For replication, we calculated the one-sided *p*-value for the direction from the longitudinal analysis using the KARE data, and used 0.05 as the significance level. Whenever the results from the two cohorts were in different directions, the *p*-values from the HEXA cohort were larger than 0.5. In Table 5 and Table 6, we added results from previous studies. If a SNP has not been significantly reported but SNPs in genes in linkage disequilibrium with it have been significantly reported, those SNPs are denoted by “*”. Table 5 and Table 6 show that GWAS using the longitudinal data in the KARE cohort identified 29 significant SNPs, 20 of which have been reported in previous GWAS, while the cross-sectional data identified only 19 genome-wide significant SNPs. Therefore we can conclude that the longitudinal data lead to substantial power improvement. In our GWAS using the longitudinal data, nine SNPs were newly detected, six of which were significantly replicated in the HEXA cohort.

**Table 4:** Correlations between different time-points for each phenotype.

Time point Phenotype	Correlation between time-points		
	1–2	2–3	1–3
SBP	0.608	0.604	0.552
DBP	0.550	0.596	0.517
GLU0	0.700	0.795	0.822
GLU120	0.675	0.748	0.715
height	0.984	0.985	0.984
BMI	0.942	0.941	0.916
HDL	0.690	0.684	0.667
log AST	0.444	0.432	0.468

Table 5 shows that rs2401887 located in CALM1 is more significantly associated with SBP in the longitudinal data analysis. GWAS of DBP identified three significant SNPs; rs3025047 in the VEGFA gene, rs7100467 near SORCS1 and rs11067763 near MED13L. It has been reported that VEGFA is related to type-2 diabetes, coronary artery disease, age-related macular degeneration and body fat [19,20,21]. For GLU0, rs12991703 located near the MARCO gene was genome-wide significant using the cross-sectional data, and rs2191346 and rs6494306, which are respectively in linkage disequilibrium with DGKB and VPS13C, were more significant using the longitudinal data.

rs7197218 in the XYLT1 gene, which is related to corneal astigmatism [22], was genome-wide significant using the cross-sectional data. rs6031492, located in GDAPL1L, is more significantly associated with GLU120 in the cross-sectional data analysis. Table 6 shows that we detected rs17178527 in AK097143 and rs11000212 in ANAPC16 as associated with BMI. rs12292858 in SIK3 was more significant using the cross-sectional data, and rs2238153 in ATXN2, rs11066280 in HECTD4 and rs183786 near ALDH1A2 were more significantly associated with HDL by longitudinal data analysis. ATXN2, HECTD4 and ALDH1A2 have been reported to have significant associations for phenotypes related to HDL [12,23,24,25,26,27,28,29,30,31,32,33,34], and the significant associations for HECTD4 and ALDH1A2 were successfully replicated in the HEXA cohort. We also performed GWAS of log-transformed AST, and Table 6 shows nine significant SNPs, rs9837421 in SH3BP5, rs10849915 in CCDC63, rs3782889 in MYL2, rs12229654 near MYL2-CUX2, rs11066280 in HECTD4, rs11066453 in OAS1, rs2072134 in OAS3, rs12483959 in PNPLA3 and rs2143571 in SAMM50. Previous studies have reported that SH3BP5, CCDC63, MYL2 and OAS3 are related to alcohol dependence phenotypes [35,36,37], and PNPLA3 and SAMM50 are related to nonalcoholic fatty liver disease [38,39], and so our results strengthen their importance in liver disease.

**Figure 4:**
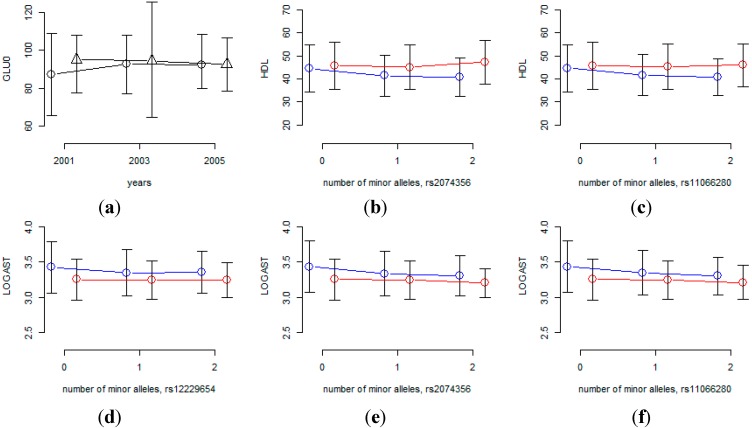
Interaction effects of SNPs with sex or time interval. (**a**) Mean of GLU0 at each time-point for each of two rs7197218 genotypes (circles indicate homozygous genotypes with no minor alleles, triangles indicate heterozygous genotypes); (**b-f**) phenotypic mean of each genotype for males and females (blue and red lines indicate male and female, respectively).

We also performed association analysis to detect gene×environment interaction, and SNPs that interact with aging, sex and time interval were identified by using the longitudinal data in the KARE cohort. Table 7 and Table 8 list SNPs with *p*-values for gene×environment interaction less than 10^−6^. Table 7 shows that rs7197218 seems to be a promising candidate SNP for interaction with aging for GLU0, and Figure 4 shows that the age effects are substantially different for this SNP. However, the MAF of rs7197218 is 0.01456, and neither it nor any other SNPs that are in linkage disequilibrium with it, were found in the HEXA cohort. Thus the significant association of this SNP could not be confirmed and it will need to be further investigated in follow-up studies. Table 8 shows that rs2074356 and rs11066280 interact significantly with sex for HDL, and rs2074356, rs11066280 and rs12229654 do so for log-transformed AST. Interestingly, rs2074356 and rs11066280 have significant interaction effects with sex for both HDL and AST. We further confirmed these significant gene×environment interactions in the HEXA cohort. Based on the direction of the coefficients for these interactions, we calculated one-sided *p*-values, and the combined *p*-values by Fisher’s and Liptak’s methods [40,41]. It has been shown that the most efficient method is achieved by Liptak’s methods if the effect sizes are expected to be the same [42]. Table 9 shows that these significant interactions were further replicated in the HEXA cohort, and the combined *p*-values become smaller. Figure 4 shows that the effects of these SNPs are substantially different for males and females and, therefore, we can conclude that the effects of these SNPs are significantly different for males and females.

In summary, we can conclude that GWAS with longitudinal data provide an efficient strategy, and our overall results show that the improvement in power is substantial, its effect being inversely proportional to ρ.

**Table 5:** Results for SBP, DBP, GLU0 and GLU120. SNPs with *p*-values less than 10^−6^ from cross-sectional or longitudinal data are listed.

SNP	Chr	Position	Nearby Gene	Minor Allele	MAF	Discovery				Replication		Previously Published
						Cross-Sectional		Longitudinal		Cross-Sectional		
						beta ± s.e	*P*	beta ± s.e	*P*	beta ± s.e	one-side *P*	
**SBP**												
rs17249754	12	88584717	ATP2B1	A	0.3732	−1.63 ± 0.27	**9.73E − 10**	−1.27 ± 0.22	**1.11E − 08**	−0.86 ± 0.49	**5.30E − 03**	Cho *et al.* NG 2009 [11]
rs11066280	12	111302166	in HECTD4	T	0.1717	−1.45 ± 0.34	2.52E − 05	−1.59 ± 0.29	**2.95E − 08**	−1.65 ± 0.43	**6.35E − 05**	Kato *et al.* NG 2011 [27]
rs2401887	14	89952963	CALM1	C	0.02125	−3.19 ± 0.93	5.89E − 04	−3.84 ± 0.78	8.58E − 07			
**DBP**												
rs10030362	4	102841866	in BANK1	C	0.2081	−0.72 ± 0.21	4.34E − 04	−0.87 ± 0.17	3.21E − 07	−0.22 ± 0.27	2.11E-01	* Zhang *et al.* Hypertension Res 2012 [43]
rs3025047	6	43854388	in VEGFA	A	0.01024	−2.65 ± 0.2	1.75E − 03	−3.64 ± 0.71	3.07E − 07			
rs7100467	10	108153198	SORCS1	T	0.02356	−2.43 ± 0.53	1.81E − 04	−2.82 ± 0.55	3.43E − 07			
rs17249754	12	88584717	ATP2B1	A	0.3732	−0.94 ± 0.17	**4.33E − 08**	−0.8 ± 0.14	**2.01E − 08**	−0.56 ± 0.23	**8.50E − 03**	Cho *et al.* NG 2009 [11]
rs11066280	12	111302166	in HECTD4	T	0.1717	−0.94 ± 0.22	1.97E − 05	−0.98 ± 0.18	**9.25E − 08**	−0.76 ± 0.38	**5.35E − 03**	Kato *et al.* NG 2011 [27]
**rs11067763**	12	114682724	MED13L	G	0.3297	−0.78 ± 0.18	1.04E − 05	−0.79 ± 0.15	**8.75E − 08**	0 ± 0.35	5.04E − 01	
**GLU0**												
**rs12991703**	2	119536716	MARCO	A	0.05655	3.62 ± 0.68	**1.18E − 07**	2.51 ± 0.58	1.55E − 05	−1.14 ± 0.7	9.11E − 01	
rs7754840	6	20769229	in CDKAL1	C	0.4761	1.8 ± 0.32	**1.72E − 08**	1.78 ± 0.27	**5.16E − 11**	0.98 ± 0.74	**3.99E − 02**	Kwak *et al.* Diabetes 2012 [44]
rs9460546	6	20771611	in CDKAL1	G	0.4808	1.75 ± 0.32	**3.38E − 08**	1.76 ± 0.27	**3.76E − 11**			
rs2191346	7	15020403	DGKB	C	0.2891	−1.72 ± 0.36	1.33E − 06	−1.53 ± 0.3	3.64E − 07	−0.62 ± 0.62	1.55E − 01	
rs6494306	15		VPS13C	A	0.3435	−1.43 ± 0.33	1.71E − 05	−1.46 ± 0.28	1.92E − 07	−1.21 ± 0.59	**2.08E − 02**	* Manning *et al.* NG 2012 [45]
**rs7197218**	16	17319136	in XYLT1	G	0.01456	7.23 ± 0.68	**1.23E − 07**	4.29 ± 1.17	2.48E − 04
**GLU120**												
rs7754840	6	20769229	in CDKAL1	C	0.4761	4.73 ± 0.78	**1.51E − 09**	4.73 ± 0.73	**1.10E − 10**			Kwak *et al.* Diabetes 2012 [44]
rs12229654	12	109898844	MYL2-CUX2	G	0.1426	−4.84 ± 1.11	1.21E − 05	−5.16 ± 1.03	5.84E − 07			Go *et al.* J Hum Genet 2013 [46]
rs2074356	12	111129784	in HECTD4	T	0.1467	−5.19 ± 1.09	2.02E − 06	−5.2 ± 1.02	3.44E − 07			Go *et al.* J Hum Genet 2013 [46]
rs6031492	20	42330963	in GDAPL1L	G	0.4949	3.84 ± 0.77	6.91E − 07	3.03 ± 0.72	2.81E − 05			
rs2868088	20	42347066	GDAPL1L	A	0.4377	−3.99 ± 0.78	2.68E − 07	−3.54 ± 0.72	1.04E − 06			

**Table 6:** Results for Height, BMI, HDL and log AST. SNPs with *p*-values less than 10^−6^ from cross-sectional or longitudinal data are listed.

SNP	Chr	Position	Nearby gene	Minor Allele	MAF	Discovery				Replication		Previously Published
						Cross-sectional		Longitudinal		Cross-Sectional		
						beta ± s.e	*P*	beta ± s.e	*P*	beta ± s.e	one-side *P*	
**Height**												
rs17038182	1	118669928	SPAG17	G	0.4188	−0.45 ± 0.08	**4.08E − 08**	−0.45 ± 0.08	**5.58E − 08**	−0.13 ± 0.13	1.53E − 01	Cho *et al.* Nat Genet 2009 [11]
rs10513137	3	142626120	in ZBTB38	A	0.2605	0.49 ± 0.09	**8.14E − 08**	0.49 ± 0.09	**5.85E − 08**	0.43 ± 0.14	**7.40E − 04**	Kim *et al.* J Hum Genet 2009 [47]
rs6918981	6	34346492	RPL35P2-NUDT3	G	0.2092	0.55 ± 0.1	**2.98E − 08**	0.55 ± 0.1	**1.72E − 08**	0.1 ± 0.15	2.51E − 01	Kim *et al.* J Hum Genet 2009 [47]
**BMI**												
**rs17178527**	6	141947773	in AK097143	A	0.2486	−0.32 ± 0.05	**2.96E − 09**	−0.31 ± 0.05	**6.35E-09**	0.05 ± 0.08	7.47E − 01	
rs11000212	10	73625658	in ANAPC16 in ASCC1	G	0.2057	0.27 ± 0.06	1.85E − 06	0.28 ± 0.06	5.14E − 07	0.05 ± 0.08	2.90E − 01	
rs9939609	16	52378028	in FTO	T	0.1262	0.34 ± 0.07	1.29E − 06	0.34 ± 0.07	7.36E − 07	0.23 ± 0.1	**1.29E − 02**	Cho *et al.* Nat Genet 2009 [11]
**HDL**												
rs271	8	19857982	in LPL	T	0.2064	1.15 ± 0.19	**4.84E − 10**	1.12 ± 0.17	**2.15E − 11**	1.81 ± 0.38	**7.70E − 07**	
rs17482753	8	19876926	LPL	T	0.1243	1.95 ± 0.23	**8.83E − 18**	1.91 ± 0.21	**1.42E − 20**	3.48 ± 0.46	**2.71E − 14**	Heid *et al.* Circ Cardiovasc Genet 2008 [48]
rs17410962	8	19892360	LPL	A	0.1244	1.95 ± 0.23	**8.25E − 18**	1.91 ± 0.21	**1.76E − 20**	3.48 ± 0.46	**2.35E − 14**	
rs12686004	9	106693247	in ABCA1	T	0.2136	−1.26 ± 0.2	**7.01E − 12**	−1.37 ± 0.17	**1.62E − 16**	−1.19 ± 0.32	**6.10E − 04**	Kim *et al.* Nat Genet 2011 [12]
rs11216126	11	116122450	BUD13	C	0.2027	1.43 ± 0.19	**2.69E − 14**	1.36 ± 0.17	**1.54E − 15**	1.44 ± 0.5	**7.45E − 05**	Kim *et al.* Nat Genet 2011 [12]
rs6589566	11	116157633	in ZNF259	C	0.2176	−1.25 ± 0.18	**1.10E − 11**	−1.15 ± 0.17	**4.47E − 12**	−1.89 ± 0.32	**8.15E − 08**	* Waterworth *et al.* Arteriosclear Thromb Vasc Biol 2010 [49]
**rs12292858**	11	116319189	in SIK3	C	0.1759	1.05 ± 0.2	**7.73E − 08**	0.88 ± 0.18	7.68E − 07	0.9 ± 0.35	**1.11E − 02**	
rs12229654	12	109898844	MYL2-CUX2	G	0.1426	−1.25 ± 0.24	**6.42E − 09**	−1.21 ± 0.2	**7.35E − 10**	−1.66 ± 0.46	**1.25E − 04**	Kim *et al.* Nat Genet 2011 [12]
rs2238153	12	110423930	in ATXN2	A	0.4579	−0.68 ± 0.16	8.76E − 06	−0.71±0.14	3.29E − 07			
**rs11066280**	12	111302166	in HECTD4	T	0.1717	−1.4 ± 0.15	**3.10E − 12**	−1.35±0.18	**1.17E − 13**	−1.92 ± 0.4	**8.95E − 07**	
rs2072134	12	111893559	in OAS3	A	0.1143	−1.39 ± 0.19	**4.53E − 09**	−1.31 ± 0.22	**1.25E − 09**	−1.36 ± 0.4	**2.33E − 03**	Kim *et al.* Nat Genet 2011 [12]
**rs183786**	15	56455402	ALDH1A2	T	0.305	−0.8 ± 0.16	8.53E − 07	−0.83 ± 0.15	**2.33E − 08**	−0.61 ± 0.33	**3.13E-02**	
rs16940212	15	56481312	LIPC	T	0.3405	1.27 ± 0.16	**1.05E − 15**	1.3 ± 0.14	**1.95E − 19**	1.11 ± 0.47	**2.04E − 04**	Kim *et al.* Nat Genet 2011 [12]
rs6494005	15	56511816	in LIPC	G	0.2678	−0.89 ± 0.2	**1.34E − 07**	−0.89 ± 0.15	**5.82E − 09**	−0.79 ± 0.39	**1.05E − 02**	
rs12708980	16	55569880	in CETP	C	0.0984	−1.67 ± 0.25	**3.63E − 11**	−1.65 ± 0.23	**5.61E − 13**	−1.88 ± 0.5	**7.40E − 05**	Kim *et al.* Nat Genet 2011 [12]
rs2156552	18	45435666	LIPG	A	0.164	−0.89 ± 0.21	1.53E − 05	−0.93 ± 0.19	5.90E − 07	−1.15 ± 0.4	**2.29E − 03**	Waterworth *et al.* Arteriosclear Thromb Vasc Biol 2010 [49]
rs4420638	19	50114786	APOC1	C	0.1121	−1.3 ± 0.16	**4.21E − 08**	−1.14 ± 0.21	**1.23E − 07**	−2.01 ± 0.47	**1.88E − 05**	Willer *et al.* Nat Genet 2013 [50]
**AST**												
rs9837421	3	15322297	in SH3BP5	G	0.193	−0.02 ± 0.01	2.14E − 04	−0.03 ± 0.01	5.79E − 07	0 ± 0	5.32E − 01	
**rs10849915**	12	109818005	in CCDC63	G	0.1758	−0.03 ± 0.01	2.00E − 06	−0.03 ± 0.01	**1.81E − 08**	−0.02 ± 0	**3.68E − 02**	
**rs3782889**	12	109835038	in MYL2	C	0.1726	−0.03 ± 0.01	3.79E − 06	−0.03 ± 0.01	**7.26E − 09**	−0.02 ± 0	**2.02E − 02**	
**rs12229654**	12	109898844	MYL2-CUX2	G	0.1426	−0.04 ± 0.01	**7.34E − 08**	−0.04 ± 0.01	**4.74E − 11**	−0.02 ± 0	**2.80E − 02**	
**rs11066280**	12	111302166	in HECTD4	T	0.1717	−0.05 ± 0.01	**8.17E − 13**	−0.05 ± 0.01	**1.70E − 18**	−0.03 ± 0	**1.94E − 04**	
rs11066453	12	111850004	in OAS1	G	0.1265	−0.03 ± 0.01	5.26E − 06	−0.03±0.01	8.72E − 07	−0.02 ± 0	7.75E − 02	
**rs2072134**	12	111893559	in OAS3	A	0.1143	−0.04 ± 0.01	**7.31E − 08**	−0.04 ± 0.01	**8.42E − 09**	−0.02 ± 0	6.75E − 02	
rs12483959	22	42657329	in PNPLA3	A	0.4157	0.03 ± 0	**1.79E − 09**	0.03 ± 0	**1.02E − 12**	0.03 ± 0	**2.14E − 06**	* Kamatani *et al.* Nat Genet 2010 [38]
rs2143571	22	42723019	in SAMM50	T	0.4136	0.02 ± 0.01	6.45E − 07	0.03 ± 0	**7.73E − 10**	0.03 ± 0	**4.22E − 05**	* Kawaguchi *et al.* PLoS One 2012 [39]

**Table 7:** Gene × environment interaction effect in the KARE cohort. Interactions of time interval with SNP were tested, and *p*-values for SNPs with genome-wide significant interaction are listed.

Effect	rs7197218 for GLU0		
	beta	Std.Error	*p*-value
SNP	5.92	1.21	1.08E − 06
time×SNP	−1.27	0.25	2.65E − 07

**Table 8:** Gene × environment interaction effect in the KARE cohort. Interactions of sex with SNPs were tested, and *p*-values for SNPs with genome-wide significant interaction are listed.

Effect	rs2074356 for HDL			rs11066280 for HDL			rs2074356 for Log(AST)			rs11066280 for Log(AST)			rs12229654 for Log(AST)		
	beta	Std.Error	*p*-value	beta	Std.Error	*p*-value	beta	Std.Error	*p*-value	beta	Std.Error	*p*-value	Beta	Std.Error	*p*-value
SNP	−4.80	0.62	7.28E − 15	−4.69	0.58	4.60E − 16	−0.13	0.02	5.52E − 13	−0.17	0.02	2.81E − 20	−0.15	0.02	1.32E − 19
sex×SNP	2.26	0.39	4.46E − 09	2.21	0.36	1.06E − 09	0.06	0.01	5.82E − 08	0.08	0.01	8.25E − 12	0.07	0.01	3.24E − 11

## 4. Discussion

It is well known that longitudinal analysis is useful to detect aging effects, and statistically efficient for detecting significant associations. In this report, we numerically calculated the sample sizes required to achieve statistical power at the genome-wide significance level, and our results showed that the power is proportionally related to the number of observations on each individual and inversely related to the correlation between the pairs of observations on an individual. In a large-scale genetic analysis, genotyping cost may be larger than the phenotyping cost, and then we can conclude that analyzing longitudinal data is an efficient strategy to improve the rate of false negative findings. However if the proportion of missing data is large, statistical power loss can be substantial; and if the missingness is not at random, even a small proportion of missing phenotypes can generate a serious bias [51]. In spite of the statistical efficiency of longitudinal data analysis, any possibility of potential bias from the missingness pattern should be carefully investigated; and it should be noted that a little carelessness can lead to a substantial bias.

**Table 9:** Combined *p*-values for gene × environment interaction. For replication, interactions of sex with SNPs were tested in the HEXA cohort and a combined *p*-value was calculated using both Fisher’s and Liptak’s methods.

Sex × SNP	HEXA Cohort *p*-value	Combined *p*-value Using Fisher’s Method	Combined *p*-value Using Liptak’s Method
rs2074356 for HDL	3.58E − 01	3.39E − 08	2.52E − 07
rs11066280 for HDL	2.184E − 01	5.37E − 09	2.52E − 08
rs2074356 for Log(AST)	4.86E − 02	5.86E − 08	4.41E − 08
rs11066280 for Log(AST)	1.23E − 02	3.13E − 12	3.10E − 12
rs12229654 for Log(AST)	7.42E − 03	7.22E − 12	4.96E − 12

Furthermore, we performed GWAS with both longitudinal and cross-sectional data, and significant results from a longitudinal data analysis in the KARE cohort were further tested in the HEXA cohort. 12 SNPs that have not been reported elsewhere were identified, and the significant *p*-values from replication studies strengthened the possibility that they are causal. In particular, GWAS with longitudinal data showed that rs3025047 is significantly associated with DBP even though it is not significantly associated in GWAS with cross-sectional data. The MAF of rs3025047 is 0.01, so it is a variant with relatively low frequency. In the HEXA cohort, rs3025047 was not available, nor were any SNPs in linkage disequilibrium with it. Even though further studies are necessary to confirm whether rs3025047 is a true causal variant, our analysis results illustrate that GWAS using longitudinal data can be an efficient strategy for rare variant association analysis.

During the last decade, more than ten thousand GWAS successfully identified disease susceptibility loci, and these findings increase our understanding of diseases. However, the so-called missing heritability [4] reveals that efficient analysis algorithms should be investigated, and GWAS of longitudinal data seem to provide a useful strategy that may bridge the gap.

## 5. Conclusions

Analyzed as a repeated measure design, the power of longitudinal data is proportionally related to the number of observations on each individual and inversely related to the correlation between the multiple observations on an individual. This facilitates finding causal SNPs and their interactions with environmental variables, as well as with age and sex. In two Korean cohorts it enabled us to find 12 novel genome-wide significant SNPs associated with eight phenotypes, and significant gene × environment interaction. Therefore, we can conclude that longitudinal data seem to provide efficient strategies for GWAS.

## Supplementary Files

Supplementary File 1

## Appendix

We assume that the null hypothesis is β = 0, and β = β*_a_* under the alternative hypothesis. According to Liu and Liang [13], the score for our hypothesis can be defined by:

(7) $$S_{\beta }=\frac{1}{{\sigma_{p}}^{2}}\sum_{i=1}^{n}{\text{X}}_{i}{1_{t}}^{\prime }{\text{R}}^{-1}({\text{Y}}_{i}-1_{t}{\text{X}}_{i}\beta -1_{t}{{\text{Z}}_{i}}^{\prime }\text{α})$$

and we denote by $\bar{\sum }$ the expected variance-covariance matrix of the score function *S_β_* under the alternative hypothesis in the absence of population substructure. Letting be the chi-square noncentrality parameter to achieve 1 – *ϕ* power at the *α* significance level, Liu and Liang [13] showed that the required sample size for the score test becomes:

(8) $$n_{a}=v/\left(\beta_{a}^{2}\bar{\sum }\right)$$

We let **1***_w_* be a *w*-dimensional column vector with elements 1. We assume that (*X_i_*, *Z_i_*) can be (*δ_l_*, *ψ_l_*), where *l* = 1, … , *L*, and define *π_l_* = *P*(*X_i_* = *δ_l_*, *Z_i_* = *ψ_l_*). If we assume ${\text{u}}_{l}=\delta_{l}1_{t}$, ${\text{D}}_{l}=\text{diag}(\psi_{l})$ is diagonal matrix, ${\text{J}}_{l}=1_{l}{1_{d}}^{\prime }$, ${\text{v}}_{l}=J_{t}D_{l}$, and *d* denotes the number of rows of ${\text{D}}_{l}$, the elements of the Fisher information matrices for β and **α** are found to be:

(9) $${\text{I}}_{\beta \alpha }^{*}=\sigma_{P}^{-2}\sum_{l}\pi_{l}{\text{u}}_{l}{\text{R}}^{-1}{\text{V}}_{l}\;\text{and}\;{\text{I}}_{\alpha \alpha }^{*}=\sigma_{P}^{-2}\sum_{l}\pi_{l}{{\text{v}}_{l}}^{\prime }{\text{R}}^{-1}{\text{v}}_{l}.$$

In the absence of population substructure, $\bar{\sum }$ can be shown to be:

(10) $$\bar{\sum }=\sigma_{P}^{-2}\sum_{l}\pi_{l}({\text{u}}_{l}^{T}-{\text{I}}_{\beta \text{α}}^{*}{{\text{I}}_{\text{αα}}^{*}}^{-1}{\text{v}}_{l}^{T}){\text{R}}^{-1}({\text{u}}_{l}-{\text{v}}_{l}{{\text{I}}_{\text{αα}}^{*}}^{-1}{{\text{I}}_{\beta \text{α}}^{*}}^{T})$$

Furthermore:

(11) $${{\text{u}}_{l}}^{\prime }{\text{R}}^{-1}{\text{v}}_{l}=\delta_{l}{1_{t}}^{\prime }{\text{R}}^{-1}{\text{J}}_{t}{\text{D}}_{l}=({1_{t}}^{\prime }{\text{R}}^{-1}1_{t})\delta_{l}({1_{d}}^{\prime }{\text{D}}_{l})$$

leads to:

(12) $${\text{I}}_{\beta \alpha }^{*}=\sigma_{P}^{-2}\sum_{l}\pi_{l}{{\text{u}}_{l}}^{\prime }{\text{R}}^{-1}{\text{v}}_{l}=\sigma_{P}^{-2}({1_{t}}^{\prime }{\text{R}}^{-1}1_{t})\sum_{l}\pi_{l}\delta_{l}({1_{d}}^{\prime }{\text{D}}_{l})$$

We let ${\text{J}}_{d}=1_{d}{1_{d}}^{\prime }$, and, because ${\text{v}}_{l}=J_{t}D_{l}=1_{t}{1_{d}}^{\prime }D_{l}$, we have:

(13) $${{\text{v}}_{l}}^{\prime }{\text{R}}^{-1}{\text{V}}_{l}={\text{D}}_{l}({{\text{J}}_{t}}^{\prime }{\text{R}}^{-1}{\text{J}}_{t}){\text{D}}_{l}=({1_{t}}^{\prime }{\text{R}}^{-1}1_{t}){\text{D}}_{l}{\text{J}}_{d}{\text{D}}_{l}$$

Thus, we have:

(14) $${\text{I}}_{\alpha \alpha }^{*}=\sigma_{P}^{-2}\sum_{l}\pi_{l}{{\text{v}}_{l}}^{\prime }{\text{R}}^{-1}{\text{v}}_{l}=\sigma_{P}^{-2}({1_{t}}^{\prime }{\text{R}}^{-1}1_{t})\sum_{l}\pi_{l}({\text{D}}_{l}{\text{J}}_{d}{\text{D}}_{l})$$

Consequently, if we let $\text{Ω}={\text{I}}_{\beta \gamma }^{*}{{\text{I}}_{\alpha \alpha }^{*}}^{-1}=(\sum_{l}\pi_{l}\delta_{l}({1_{d}}^{\prime }{\text{D}}_{l})){\left(\sum_{l}\pi_{l}({\text{D}}_{l}{\text{J}}_{d}{\text{D}}_{l})\right)}^{-1}$, some tedious algebraic manipulations lead to:

(15) $$\bar{\sum }=\sigma_{P}^{-2}\sum_{l}\pi_{l}({\text{u}}_{l}^{T}-{\text{I}}_{\beta \text{α}}^{*}{\text{I}}_{\text{αα}}^{*}{\text{v}}_{l}^{T}){\text{R}}^{-1}({\text{u}}_{l}-{\text{v}}_{l}{{\text{I}}_{\text{αα}}^{*}}^{-1}{{\text{I}}_{\beta \text{α}}^{*}}^{T})=\sigma_{P}^{-2}\sum_{l}\pi_{l}(\delta_{l}^{2}({1_{t}}^{\prime }{\text{R}}^{-1}1_{t})-2\delta_{l}{\text{R}}^{-1}\text{J}{\text{D}}_{l}\text{Ω}\prime +\text{Ω}{{\text{D}}_{l}}^{\prime }\text{J}\prime {\text{R}}^{-1}\text{J}{\text{D}}_{l}\text{Ω}\prime )\;=\sigma_{P}^{-2}({1_{t}}^{\prime }{\text{R}}^{-1}1_{t})\sum_{l}\pi_{l}(\delta_{l}^{2}-2\delta_{l}{1_{d}}^{\prime }{\text{D}}_{l}\text{Ω}\prime +\text{Ω}{\text{D}}_{l}{\text{J}}_{d}\text{Ω}\prime )$$

If we denote $\sum_{l}\pi_{l}(\delta_{l}^{2}-2\delta_{l}{1_{d}}^{\prime }{\text{D}}_{l}\text{Ω}\prime +\text{Ω}{\text{D}}_{l}{\text{J}}_{d}\text{Ω}\prime )$ by *K*, the required sample size becomes:

(16) $$n_{a}=v/\left(\beta_{a}^{2}\bar{\sum }\right)=\frac{{\left(z_{1-\alpha /2}+z_{1-\phi }\right)}^{2}\sigma_{P}^{2}}{K\beta_{a}^{2}}\left(\frac{1+(t-1)\rho }{t}\right)$$

because ${1_{t}}^{\prime }{\text{R}}_{-1}1_{t}=t/(1+(t-1)\rho )$. This completes the derivation for *n_α_*.
