# Supplementary material for: On the Analysis of a Repeated Measure Design in Genome-Wide Association Analysis
Source: Int J Environ Res Public Health. 2014 Nov 28;11(12):12283–303. doi: 10.3390/ijerph111212283 (PMC4276614; doi:10.3390/ijerph111212283)
Supplement: Supplementary File 1 [file ijerph-11-12283-s001.pdf]

Supplementary Information

On the Analysis of a Repeated Measure Design in Genome-Wide Association Analysis

---

Figure S1. Q-Q plots for eight phenotypes with the results from longitudinal data analysis.

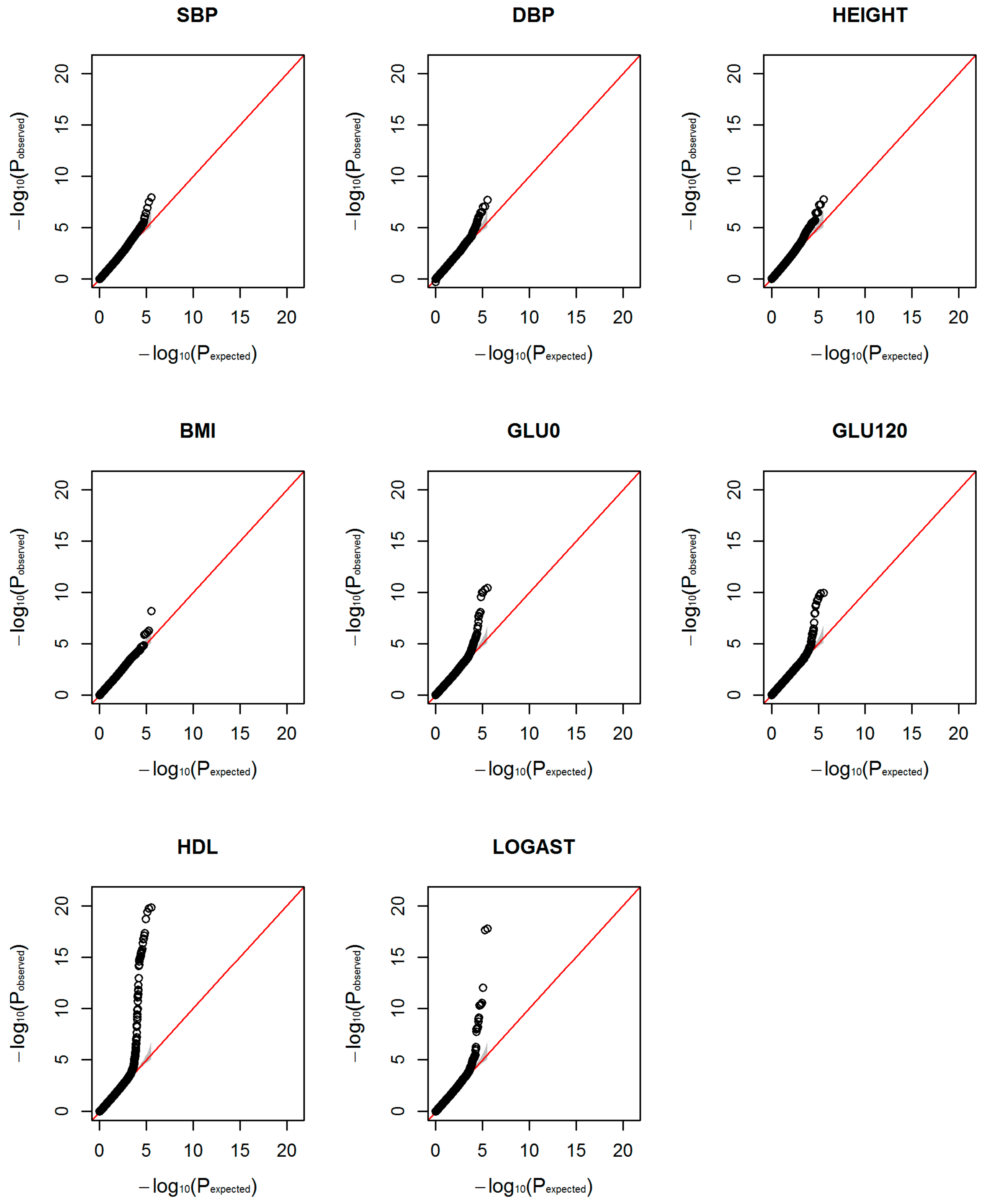

Figure S2. Q-Q plots for eight phenotypes with the results from cross-sectional data analysis.

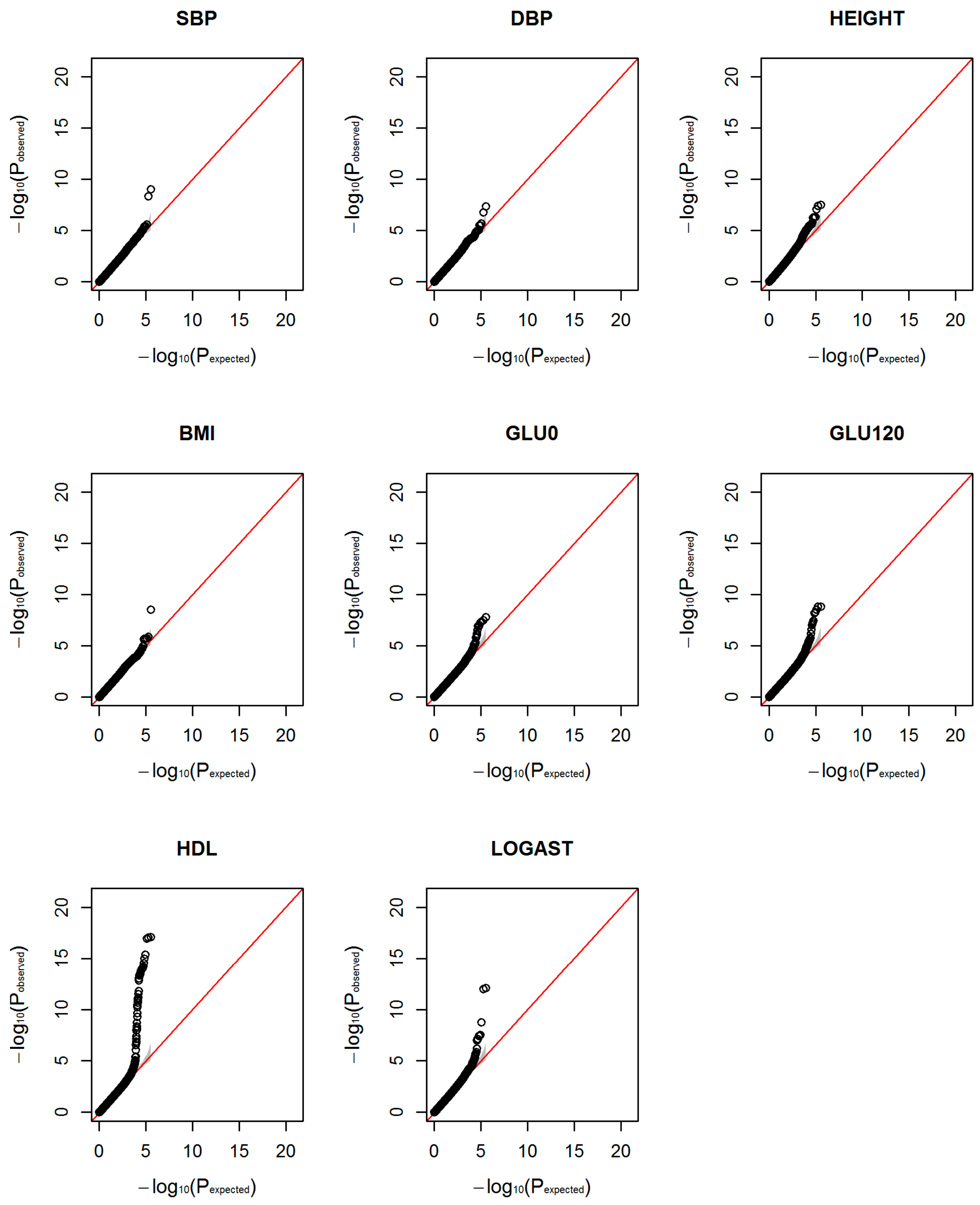

Figure S3. Manhattan plots for eight phenotypes with the results from longitudinal data analysis.

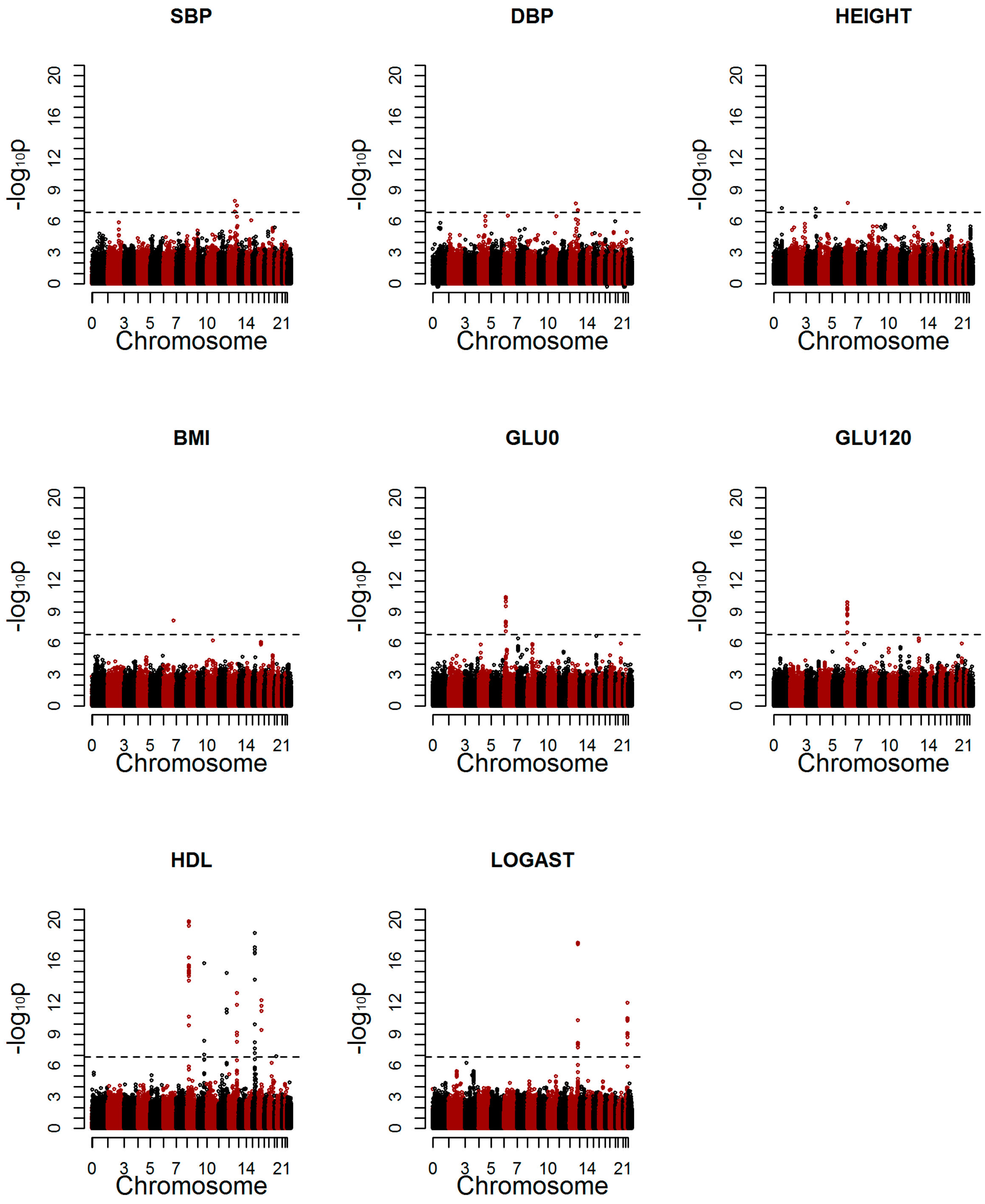

Figure S4. Manhattan plots for eight phenotypes with the results from cross-sectional data analysis.

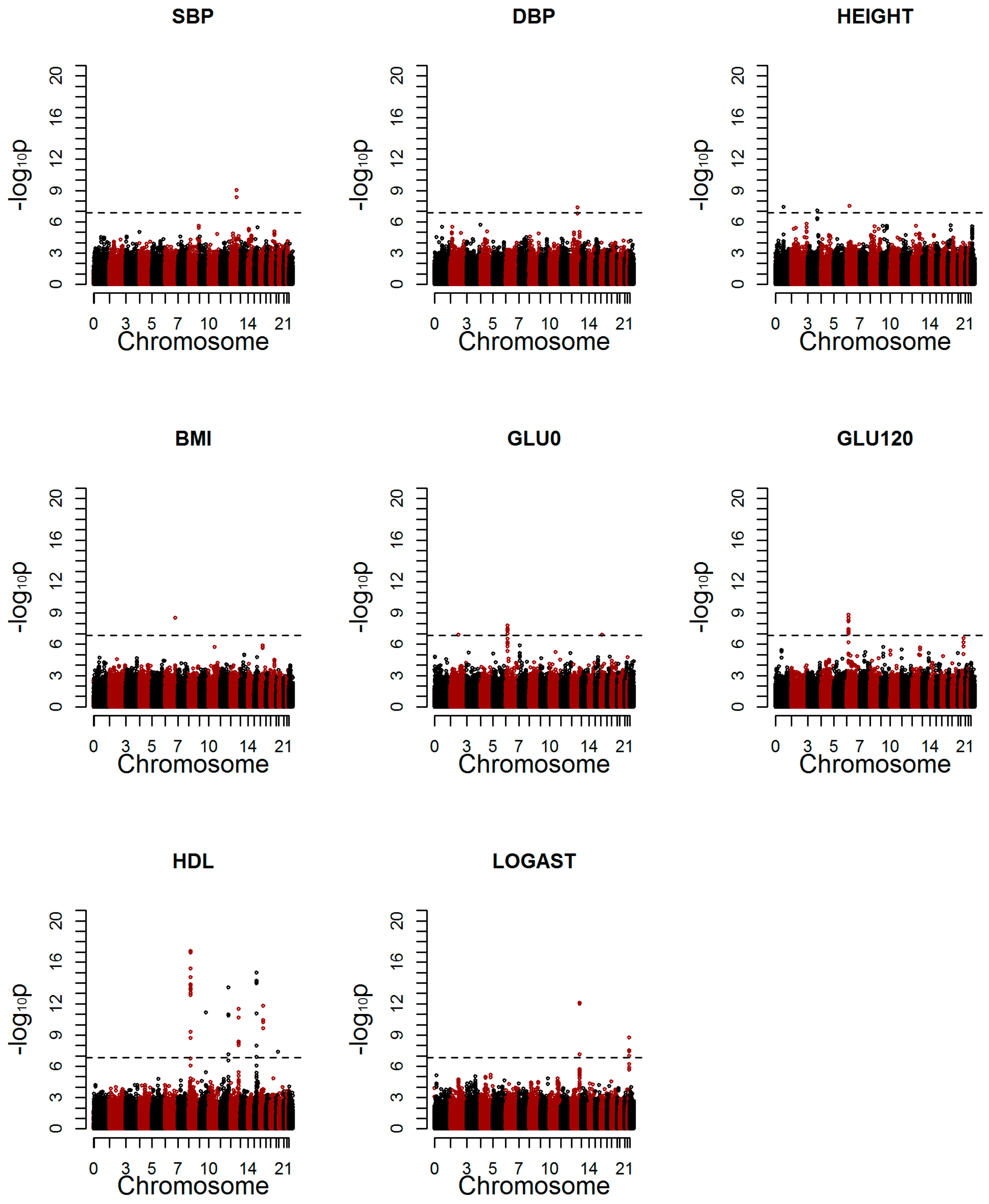

© 2014 by the authors; licensee MDPI, Basel, Switzerland. This article is an open access article distributed under the terms and conditions of the Creative Commons Attribution license (http://creativecommons.org/licenses/by/4.0/).
